# Digital implant placement accuracy: a clinical study on a fully-guided flapless single-unit immediate-loading protocol

**DOI:** 10.1186/s40902-023-00387-5

**Published:** 2023-05-17

**Authors:** Parsa Pirooz, Faezeh Atri, Paria Gholami, Mohammad Bayat

**Affiliations:** 1 School of Dentistry, Tehran University of Medical Sciences, Tehran, Iran; 2 Department of Prosthodontics, School of Dentistry, Tehran University of Medical Sciences, Tehran, Iran; 3 Department of Oral and Maxillofacial Surgery, School of Dentistry, Tehran University of Medical Sciences, Tehran, Iran

**Keywords:** Single-tooth dental implants, Immediate dental implant loading, Computer-assisted surgery, Computer-aided design

## Abstract

**Aims:**

The primary aim of the present study was to measure the discrepancy between the virtual and the actual position of the single-unit implants placed via a digitally-designed fully-guided surgical template using a flapless surgical technique. Prefabricated provisional restorations and periodontal factors were evaluated after the immediate loading of implants and 3 months after the surgery, respectively.

**Materials and methods:**

Fourteen implants in nine patients were virtually planned after importing intraoral scans and cone-beam computed tomography (CBCT) records into 3D planning software. Accordingly, fully-guided surgical templates, customized abutments, and provisional restorations were designed and fabricated. The implant position after the surgery was compared with its virtual counterpart in terms of angular and apical linear deviations. Implants were immediately loaded after the surgery, and the occlusal level of the delivered provisional restorations was compared with their designed positions. Early implant failure, bleeding on probing, and peri-implant pockets were documented on the 3-month follow-up.

**Results:**

A mean angular deviation of 5.07 ± 2.06° and a mean apical linear deviation of 1.74 ± 0.63 mm resulted. Two out of 14 implants failed within the first 3 months of the surgery, and the occlusal level difference was calculated for nine prefabricated provisional restorations.

**Conclusions:**

DIONAVI protocol has been evaluated regarding its accuracy, and an estimation of the expected deviation is presented to the clinicians using this protocol. However, before widespread use, immediate-loading protocols and provisional restorations must be studied further.

**Trial registration:**

IRCT, IRCT20211208053334N1. Registered 6 August 2022.

## Introduction

Since the advent of implant-supported prostheses, clinicians and manufacturers have constantly strived to produce more acceptable outcomes. Considering surgical and prosthetic aspects of the implant placement, it has evolved from the two-staged delayed-loading flapped technique being the prevalent treatment choice to implementing immediate loading, flapless, and digitally-guided techniques into the routine mode of care.

Flapless implant placement has shown several merits, such as decreased morbidity, less patient discomfort, shorter clinical time, reduced post-op bone loss, and improved blood microcirculation [1–9]. Nevertheless, this technique comes with its disadvantages, including poor real-time visualization of the implant site, which demands accurate preoperative planning to prevent the violation of vital structures and bone perforations [6]. Implant placement accuracy using the flapless technique was found unacceptable while employing conventional 2D radiographs in conjunction with a regular examination of the implant site [10].

Computer-guided surgery has been introduced in an attempt to reach a more optimized implant positioning. Recent advancements in imaging modalities (e.g., the introduction of cone-beam computed tomography (CBCT) and digital scanners) and the production of sophisticated computer programs laid the foundations for computer-guided surgery [11]. The level of guidance (partially or fully) and the surgeon’s ability to modify the implant’s position during the surgery (static or dynamic) are two features by which the guided surgery is classified [12]. In line, a meta-analysis has shown that static computer-aided implant placement’s accuracy is superior to free-handed (no guides included) and partially-guided implant placement (regardless of the flapped or flapless approach) [13].

The flapless surgery’s combination with the computer guidance was the answer to concerns about its accuracy. Comparing flapless static computer-aided with free-handed and partially-guided implant placement in terms of accuracy shows angular and linear apical deviation significantly lower in the former group [14–18].

Immediate-loading protocols have become more popular due to their ability to improve the patient's experience, reduce edentulous periods, satisfy esthetic demands, and preserve gingival architecture [19, 20]. However, these protocols present complications; for instance, they have been linked with more implant failure compared to the delayed-loading treatment plan, which means proper case selection is vital [19].

Whereas several studies evaluated flapless techniques, computer-guided surgery, and immediate-loading procedures individually, it is still necessary to research the therapeutic procedures that combine these techniques into a whole treatment plan. Moreover, this study employed a relatively modern technique for designing surgical templates (which uses CBCT records and intraoral scans), in contrast to the conventional dual scan technique with CBCT records of radiographic templates used in the previous studies.

This clinical study primarily aimed to evaluate the implant placement accuracy of a flapless computer-guided single-unit protocol by comparing the virtually planned and the actual post-surgical position of the implant fixture (by assessing angular deviation and linear apical deviation). A 3-month follow-up was performed following immediate-loading of the implants. The difference between the virtual and the delivered provisional restorations’ occlusal level, early implant failure, bleeding on probing, and peri-implant pockets were secondary outcome variables evaluated in this study.

## Materials and methods

Referred patients to the dental clinic of Tehran University of Medical Sciences were examined and selected based on the inclusion and exclusion criteria. Nine patients — seven males and two females — with a mean age of 52 years and 14 single-unit implant sites were included in the study. An oral and maxillofacial surgeon (M.B) with over 20 years of experience carried out all surgical operations (fully-guided flapless single-unit implant placement). Subsequently, a senior dental student (P.P) familiar with the digital scanner performed intraoral scans and delivered provisional restorations under the prosthodontist’s (F.A) supervision. Tehran University of Medical Sciences ethical committee approved this study before any clinical phase started (approval ID: IR.TUMS.MEDICINE.REC.1400.1101). Informed consent was obtained before patients' enrolment in the study.

### Inclusion and exclusion riteria

The study’s target group was partially edentulous patients who lost their teeth at least 2 months before the surgery. They were also candidates to receive single-unit final restorations with at least one adjacent tooth present. Moreover, patients had to have sufficient bone and not require graft or sinus lift surgery at the implant site. Patients had no systemic or local diseases contradicting oral surgery and no signs of parafunctional habits or temporomandibular disorders. Patients with poor oral hygiene, smoking habit (> 10 cigarettes per day), and any active periodontal infections were excluded as well.

### Sample size

The software used for sample size calculation was PASS 15 (NCSS LLC, TX, USA), and the formula was a one-sample *t*-test. The primary outcome variables (angular and linear apical deviation) were considered for sample size calculation. Valente et al. [21] obtained 1.6 ± 1.2 mm for linear apical deviation and 7.9 ± 4.7° for angular deviation in a similar study. Consistent with the mentioned study, a sample size suitable to find a mean angular deviation of more than 4° and a mean linear apical deviation of more than 1 mm was calculated (alpha was set to 0.05, target power was set to 80%). The calculated sample size for the angular and linear apical deviation were 13 and 14 samples, respectively. Therefore, 14 implants were included in the current study.

### Pre-surgical phase

**Fig. 1:**
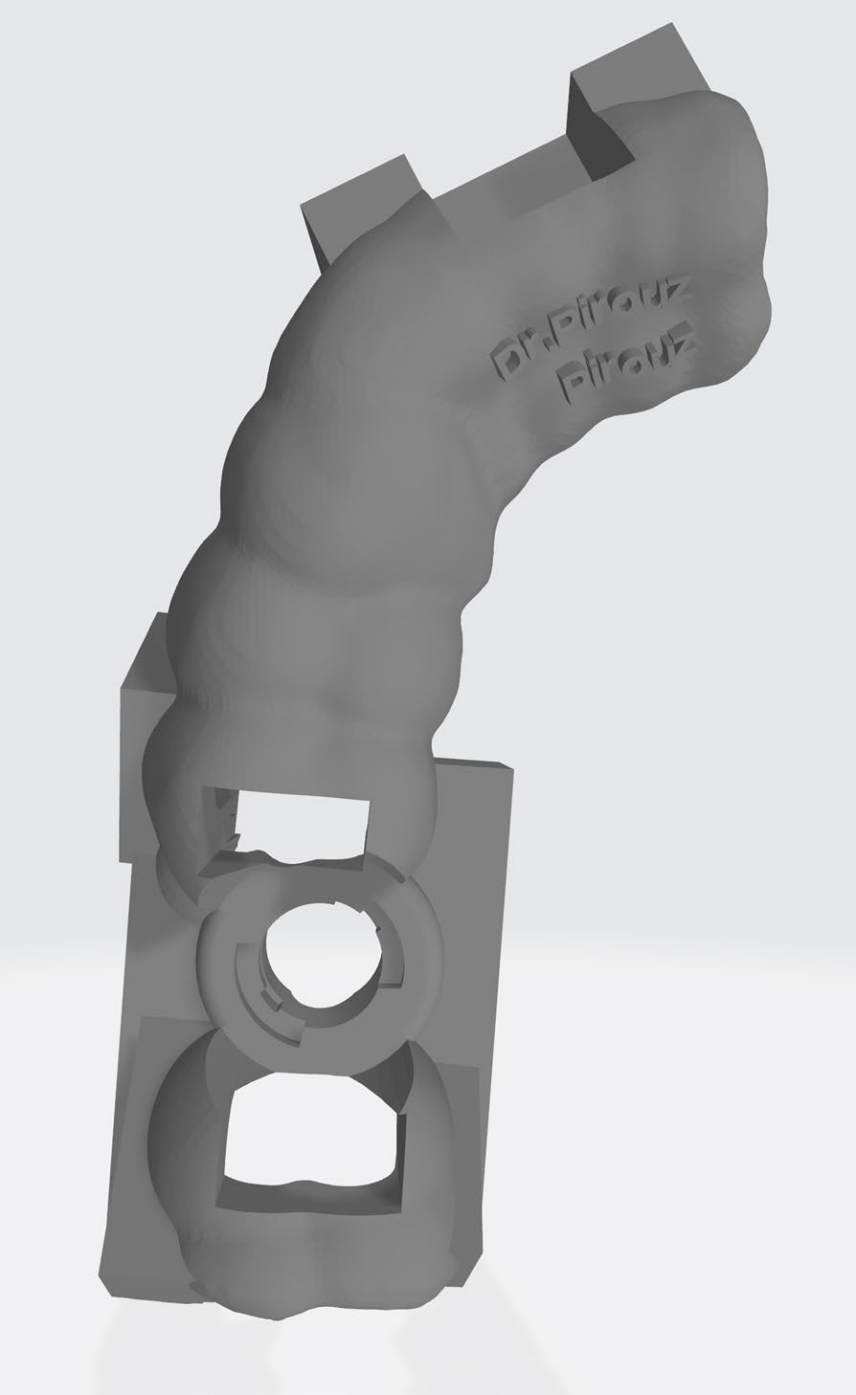
Standard Tessellation Language (STL) file of the designed surgical template generated by the Implant Studio software
  A customized abutment and a provisional restoration were designed by importing the template’s STL file into TRIOS Design Studio software (3Shape Global, Copenhagen, Denmark) (Fig. 2). Designing these restorative parts took place in adherence to the following principles: No occlusal contact with the opposing teeth, proper proximal contact with the adjacent teeth, marginal adaptation to the customized abutment, and presence of an access hole on the restoration for screw handling [19]. Titanium customized abutments were milled by a milling machine (Programill PM7, DIO Implant, Busan, South Korea), and provisional restorations were printed by the mentioned 3D printer using polymethylmethacrylate blocks (Inlab Dental PMMA Block, Dentsply Sirona, Charlotte, USA) [19].

1. A CBCT (WhiteFox, Acteon, Roma, Italy) covering both jaws was obtained, and a DICOM (Digital Imaging and Communications in Medicine) file was exported. An intraoral scan (Medit i500, Medit, Seoul, South Korea) of both jaws was also performed (after calibration of the scanner), and two STL (Standard Tessellation Language) files were generated (each representing a jaw).
2. A single-sleeved fully-guided tooth-supported template was designed by importing and superimposing DICOM and STL files into Implant Studio software (3shape Global, Copenhagen, Denmark) [22]. Optimized final restoration position (as a preliminary design of the restoration appears on the screen) and avoiding vital anatomical structures were basic principles for the virtual positioning of the implants. The template’s STL file was exported and sent for 3D printing (DIO PROBO, DIO Implant, Busan, South Korea) after the approval of the surgeon and prosthodontist (Fig. 1).

**Fig. 2:**
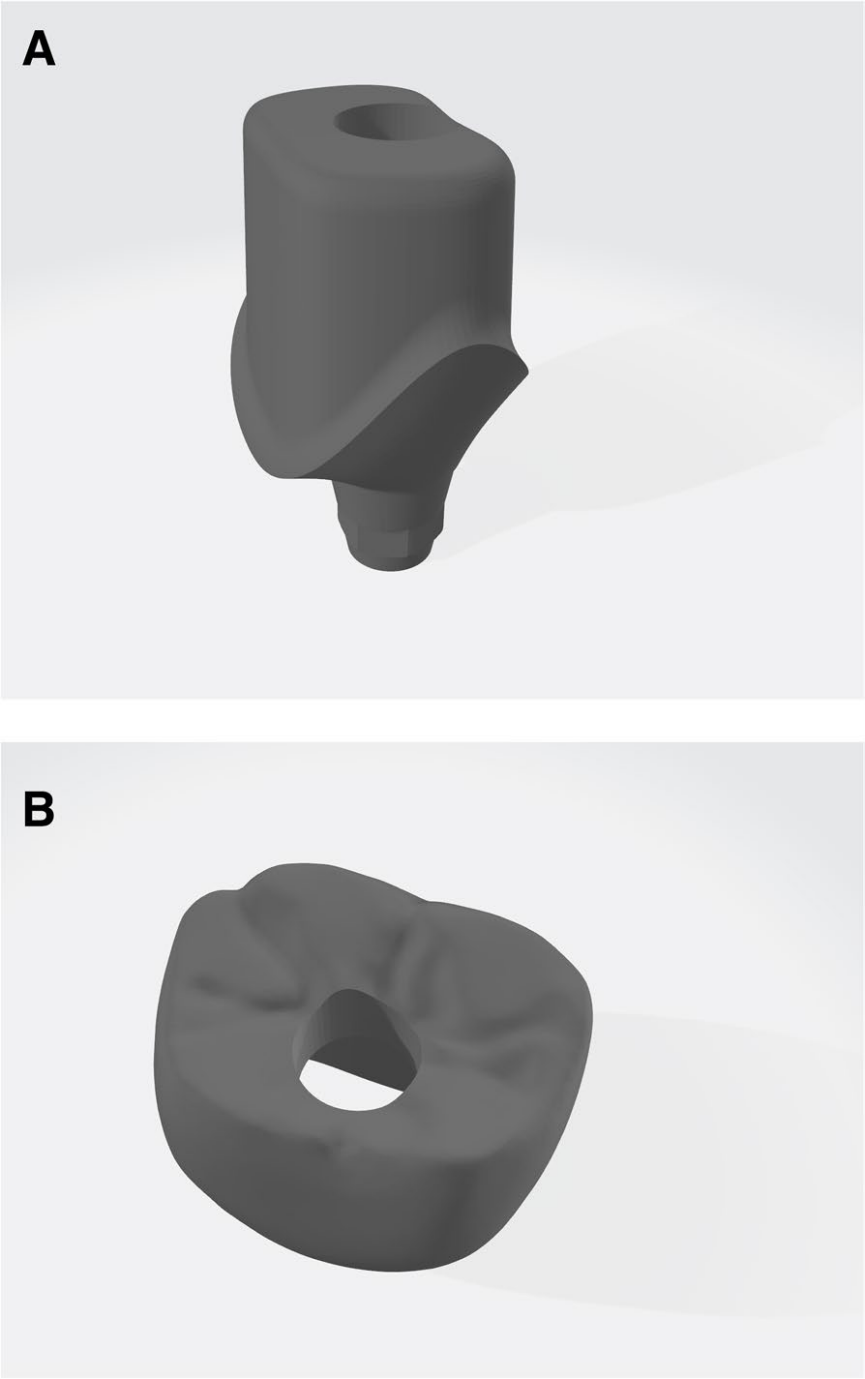
Standard Tessellation Language (STL) files of the customized abutment (**A**) and the provisional restoration (**B**) generated by the TRIOS Design Studio software
3. Surgical templates, customized abutments, and provisional restorations were sterilized with ethylene oxide before packing them.

### Surgical phase

**Fig. 3:**
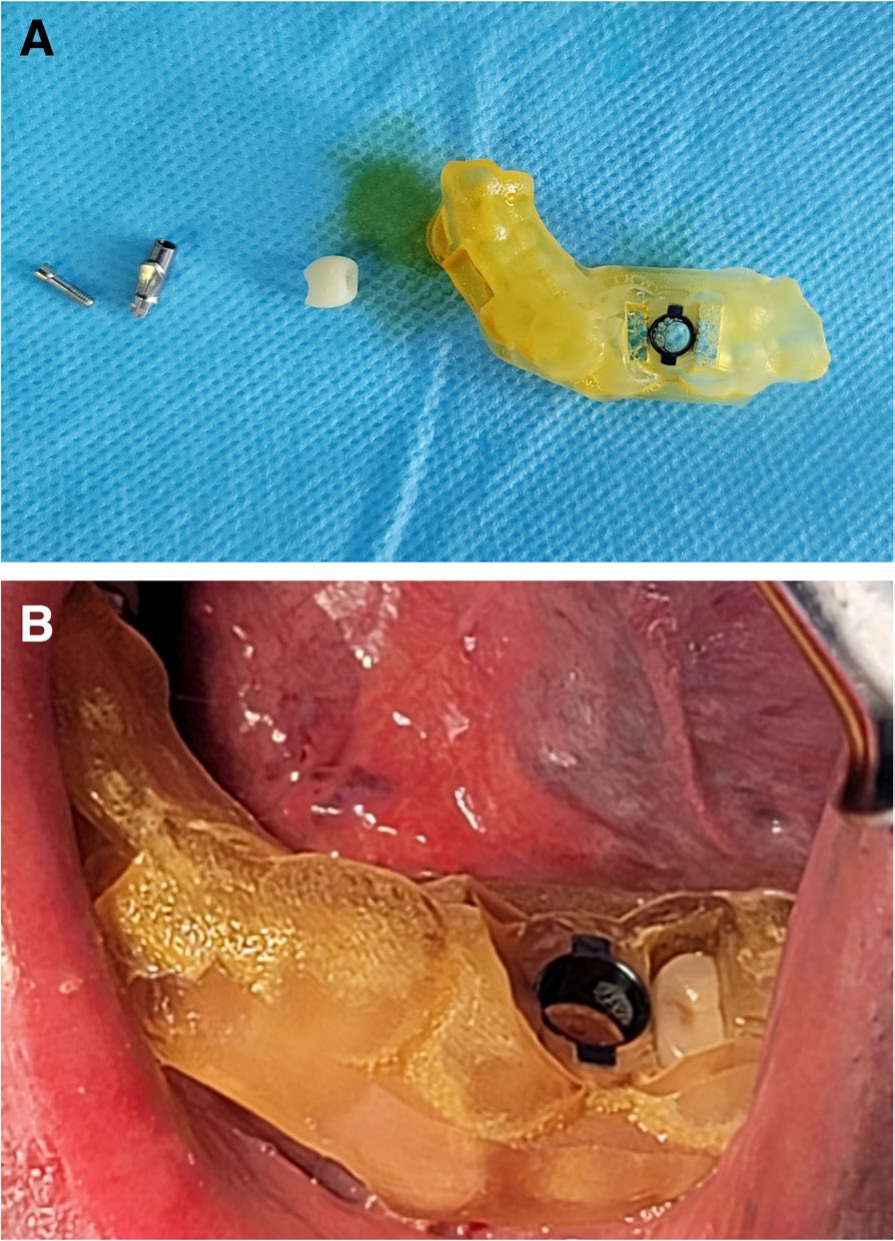
**A** From right to left: Surgical template, provisional restoration, customized abutment, and abutment screw. **B** The surgical template is inserted and stabilized in the patient’s mouth

Surgeries were carried out in an outpatient environment. The patient was prepared using the aseptic technique. After the patient rinsed his mouth with 0.2% chlorohexidine mouthwash (Chlorohexidine-Najo 2%, Iran Najo, Tehran, Iran), local anesthesia was obtained by injecting 2% lidocaine with 1:100,000 (Xylopen 2%, Exir, Tehran, Iran). The surgical template was soaked in betadine solution (Povidone Iodine 10%, Aburaihan Pharmaceutics, Tehran, Iran) before inserting and checking its stability (Fig. 3). Subsequently, the surgeon punched out the covering soft tissue and prepared the surgical site employing the DIONAVI implant system drilling protocol [22]. The surgeon inserted the implant fixture (DIO UF(II), DIO Implant, Busan, South Korea) and its customized abutment immediately afterward.

### Post-surgical phase

The patient was prescribed amoxicillin 500 mg (Amorax, Daanapharma, Tehran, Iran) 3 times daily for 7 days after discharge. A second CBCT record was obtained immediately after the surgery. The implant fixture was loaded immediately after the surgery by cementing the respective provisional restoration to the customized abutment using Zinc phosphate cement (Hoffmannʼs Zinc Phosphate Cement, Hoffmann Dental Manufacturer, Berlin, Germany). Eventually, the occlusal surface was adjusted to obtain 1 mm clearance to the opposing teeth. Another intraoral scan was taken after the cementation and adjustment process.

### Three-month follow-up

To immediately resolve and record any potential surgical or prosthetic complications during the following 3 months, patients were instructed to notify the surgeon if any progressive pain, fixture movement, swelling, puss discharge, or chewing difficulties were present. The fixture was extracted in case of the early implant failure (clinically mobile and radiolucency around the fixture present in a periapical radiograph) [23]. On the follow-up session 3 months after the surgery, patients were examined for the presence of bleeding on probing and peri-implant pockets (> 3 mm probing depth at the buccal or lingual surfaces, > 4 mm probing depth at interproximal surfaces). The periapical radiograph (62 kVp/8 mA) was also recorded to ensure sufficient osteointegration using a digital intraoral imaging system (DIGORA Optime, DEXIS, Brea, USA).

### Accuracy analysis

In order to compare the positions of the virtually planned and the actual implant fixture, a second surgical template was designed. Post-surgical intraoral scan and CBCT records were imported and superimposed in the TRIOS designing software. Second surgical templates were designed using the same principles and in a similar structure as those used for the surgeries, with only one difference present: The virtual fixture outline matched with the actual fixture outline in the post-surgical CBCT.

**Fig. 4:**
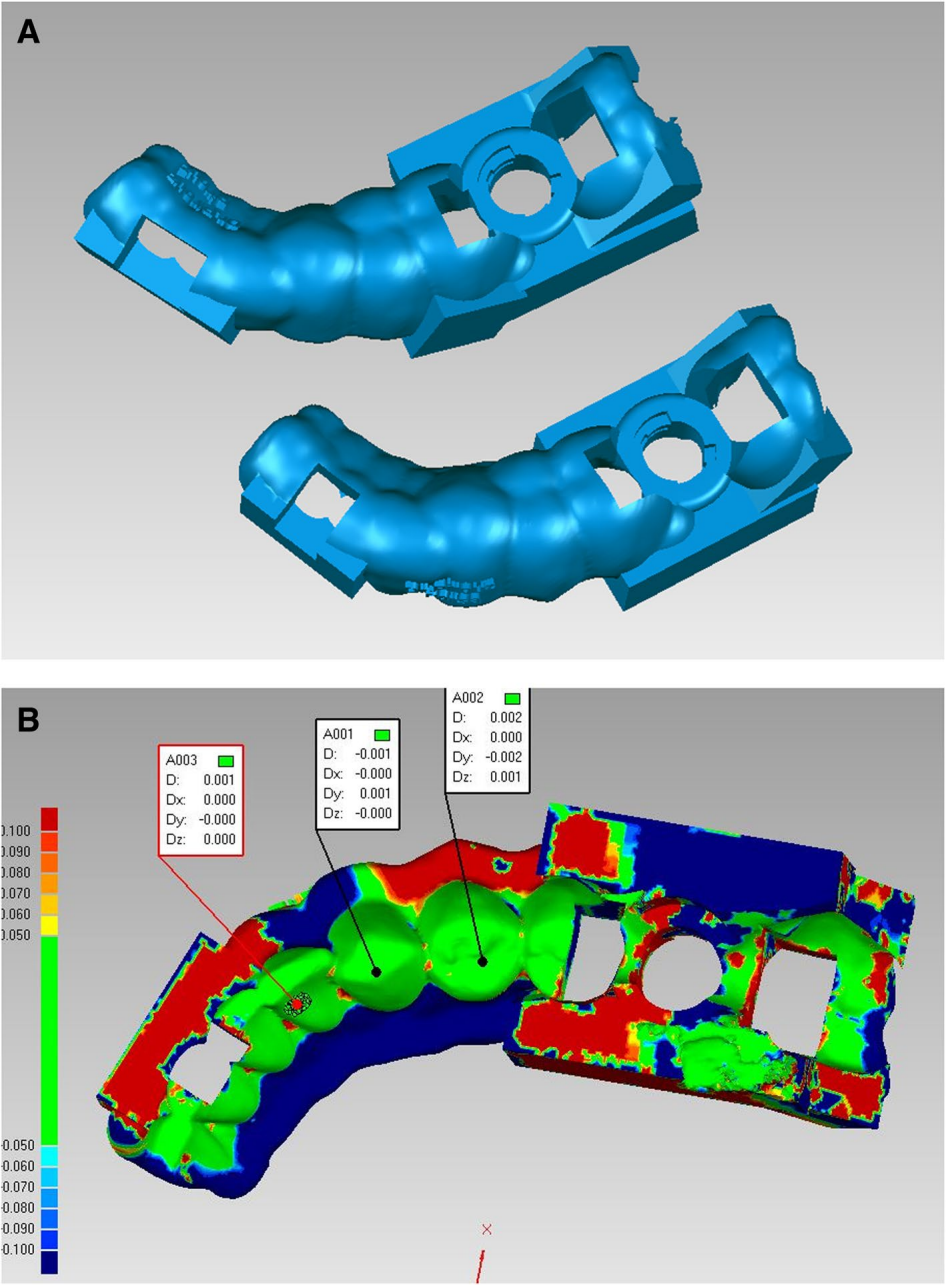
**A** Initial and secondary surgical templates are imported into the Control X software. **B** Two templates are superimposed using the templates’ inner surfaces

**Fig. 5:**
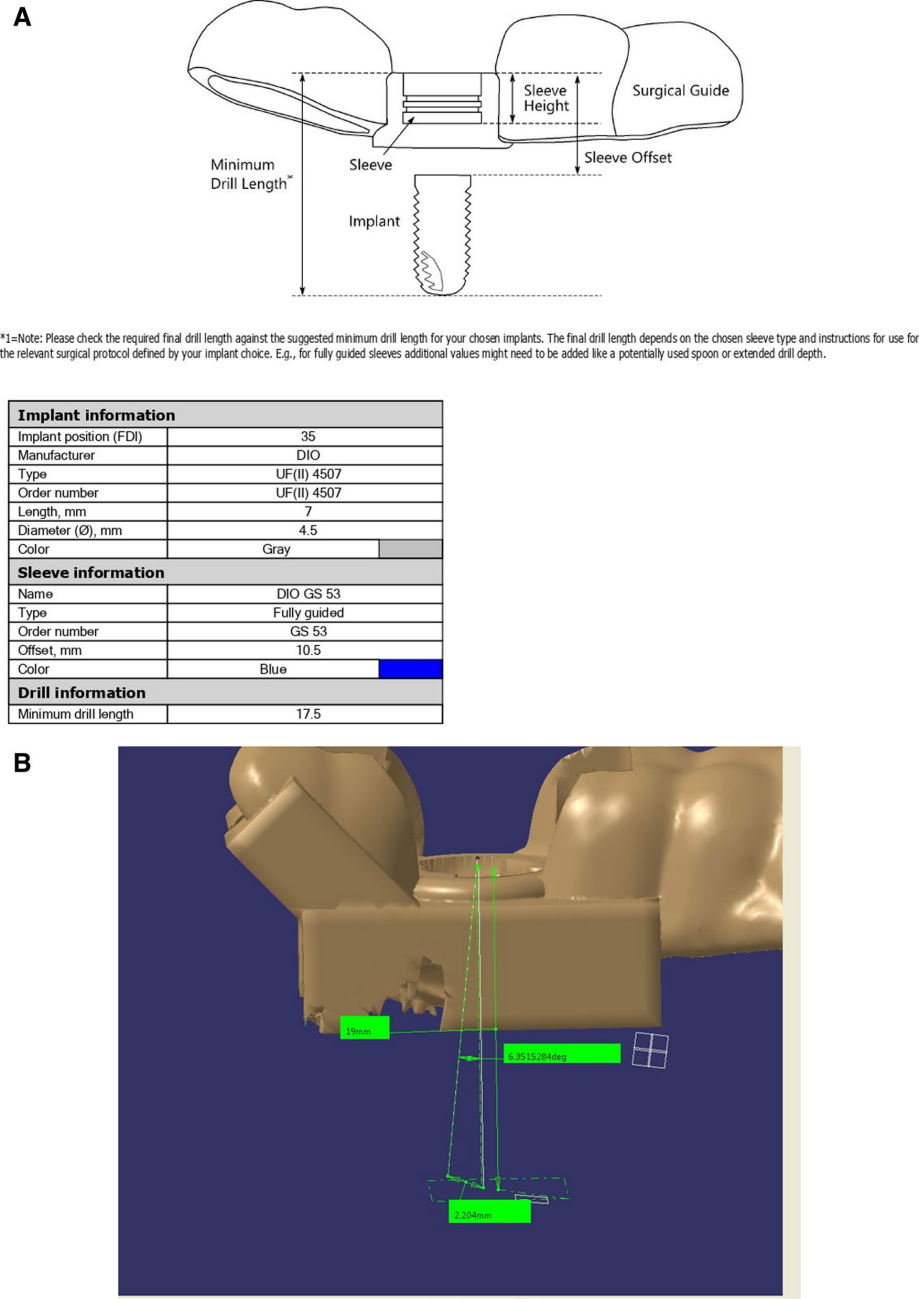
**A** The drilling protocol is automatically generated alongside the surgical template’s STL file, which contains minimum drill length and other related information. **B** Angular and apical linear deviation is calculated after drawing implant axes and locating implant apexes

The initial template design was imported into the Control X software (Geomagic Inc., Raleigh, United States). After aligning the template in relation to the Cartesian coordinate axes (X, Y, and Z), the post-op template design was imported. These two designs were superimposed by matching the inner surface of the templates, which are negative duplicates of the implant site's adjacent teeth (Fig. 4). Considering the sleeve’s cylindrical shape, the drilling axis of each template (which is equal to the implant axis) was drawn by connecting centers of top and bottom circular bases. The angle between the implant axes of these two templates is the angular deviation. The implant apex was located on the drilling axis at a “minimum drill length” distance of the top circular basis of the sleeve’s cylindrical shape. The linear apical deviation was calculated by measuring the distance between the initial template’s and the post-op template’s apex locations (Fig. 5).

**Fig. 6:**
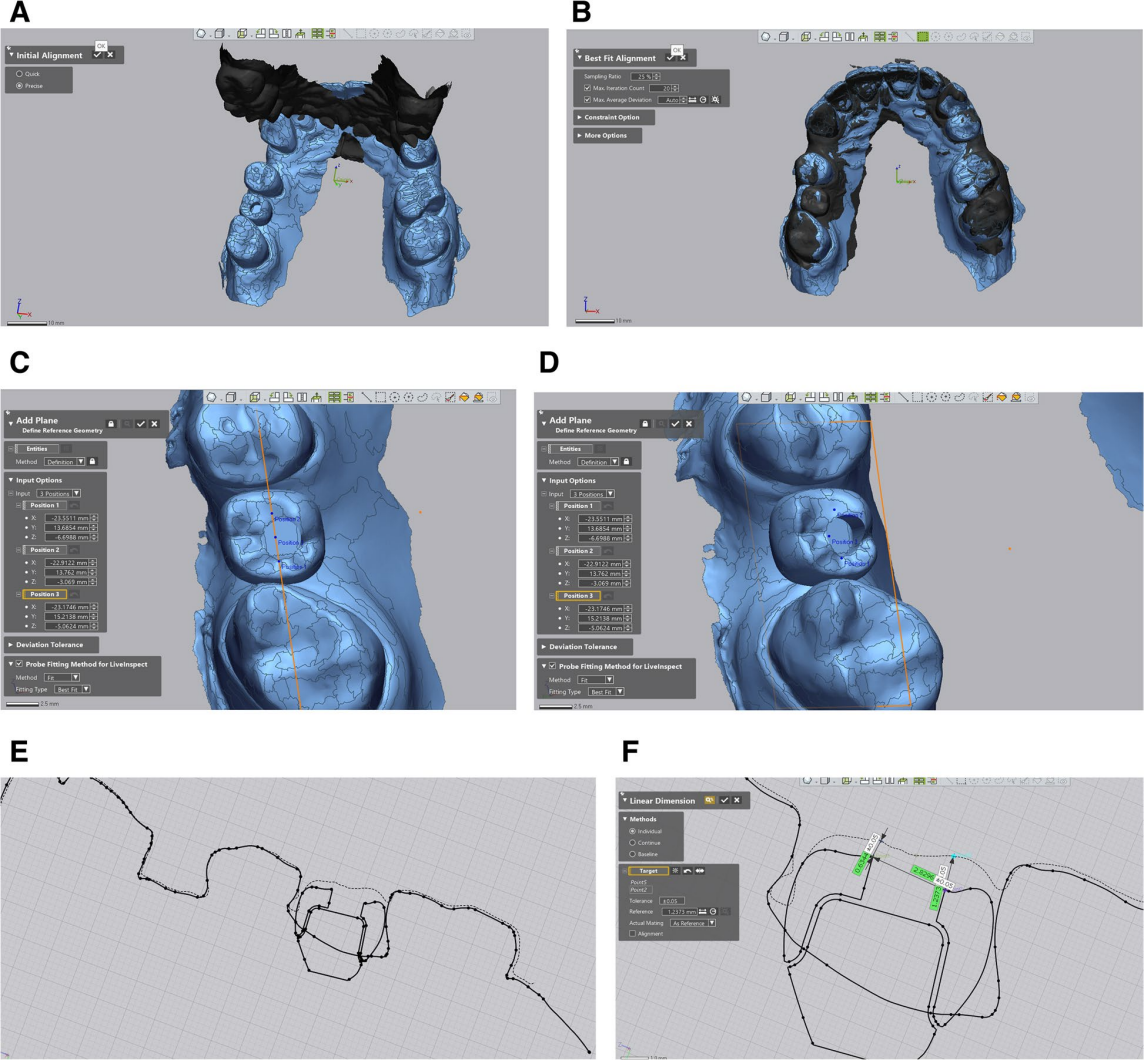
The occlusal level difference is calculated in the following steps: **A** Standard Tessellation Language (STL) files of the initial (containing the provisional restoration design) and the second intraoral scans are imported into the Control X software. **B** Two scan files are superimposed onto each other by the software’s initial and best fit features. **C**, **D** A plane roughly crossing the central groove of the virtual restoration (and parallel to its access hole axis) is constructed by the “3 position” feature of the software. **E**, **F** A 2D cross-section of the plane is generated, and the occlusal level difference is measured between the middle points of the designed and actual crowns

Measuring the difference between the occlusal level of the virtually planned restoration and the outcome (after clinical occlusal adjustment) became possible by importing and superimposing the initial intraoral scan containing the virtual restoration and the second intraoral scan into the Control X software (Using the Initial and Best Fit Alignment). A plane roughly crossing the virtual restoration’s central groove (incisal edge in case of the anterior teeth) and parallel to the virtual restoration’s access hole was constructed. A 2D cross-section of the plane was generated; Subsequently, the distance between the middle points of the designed and actual crowns was determined and recorded as the occlusal level difference (Fig. 6).

## Results

A total of 14 implants were placed for nine patients. Comparison between the virtual and actual implant positions showed a mean angular deviation of 5.07° (standard deviation [SD] = 2.06°, minimum [min] = 1.77°, maximum [max] = 9.73) and a mean linear apical deviation of 1.74 mm (SD = 0.63 mm, min = 0.94 mm, max = 2.75 mm) (Table 1).

**Table 1:** Implant treatment outcome in terms of placement accuracy (*AD* angular deviation, *ALD* apical linear deviation, *OLD* occlusal level difference) and 3-month follow-up examination (*EIF* early implant failure, *BOP* bleeding on probing, *PP* peri-implant pocket)

	**Tooth number**	**Fixture dimensions (diameter/length) (mm)**	**AD (°)**	**ALD (mm)**	**OD (mm)**	**EIF (+ / −)**	**BOP (+ / −)**	**PP (+ / −)**
**Upper posterior**	16	4.5/10	3.99	1.41	NA	−	NA	NA
	26	4.5/10	1.77	0.94	NA	−	NA	NA
	25	4/8.5	4.69	1.72	NA	+	NA	NA
	16	4.5/7	2.90	1.09	0.9	−	−	−
**Lower posterior**	36	4/10	6.35	2.2	0.8	−	+	−
	36	4.5/8.5	6.22	0.98	NA	−	−	−
	46	4.5/8.5	3.26	2.75	NA	+	NA	NA
	46	4.5/10	4.51	1.18	< 0.1	−	−	−
	37	4.5/7	9.73	2.66	< 0.1	−	+	−
	47	4.5/7	7.35	2.48	< 0.1	−	−	−
	36	4/10	5.99	1.42	< 0.1	−	−	−
	46	4.5/11.5	3.36	1.62	0.4	−	−	−
	35	4.5/7	4.81	2.25	< 0.1	−	+	−
**Upper anterior**	22	3.3/13	6.02	1.61	1.5	−	−	−
**Mean**			5.07	1.74				
**SD**^**a**^** ( ±)**			2.06	0.63				

^a^*SD* standard deviation

Nine provisional restorations were analyzed for the occlusal level difference, as five were missed due to the following reasons: One implant failed before the second intraoral scan, two were unacceptable for immediate loading due to insufficient primary stability, and two restorations were not adjusted as no opposing teeth were present (Table 1).

Two implants were subject to early failure within less than a month of the surgery. As for other complications during the first 3 months of the surgery, one screw loosening and one restoration breakdown were reported. They were resolved immediately by screw tightening and cementing a duplicate of the provisional restoration, respectively. In the 3-month follow-up examination, ten remaining restorations were examined for Bleeding on Probing and peri-implant pockets (Table 1).

## Discussion

There are a limited number of studies evaluating the accuracy of implant placement by digitally-designed surgical templates in which CBCT and intraoral scans are the input data. Still, we need to investigate different designing and surgical protocols to form a comprehensive opinion. Regarding the present study, the accuracy measurement was successfully done without any dropouts. However, secondary outcome variables were reported for some cases due to the presented complications. A digital workflow and tools required for accuracy measurement were described, which can be drawn on in future similar studies.

Guided surgery was introduced more than three decades ago as a manner to translate implant planning into the real surgical setting. Acrylic surgical templates — designed using optimal positioning of implant-supported prosthesis on stone casts — served as the conventional mean for guided surgery [24]. CBCT radiographs and stereolithographic techniques made it possible to virtually plan and fabricate surgical guides without including casts [25]. Virtual planning became widespread with the advent of dual scan protocols, which employed CBCT imaging of radiopaque templates to locate suitable implant positions in the jaw [26].

Several studies evaluated the accuracy of surgical templates fabricated by the dual scan protocol [15, 17, 21, 27–30]. Most recently, Magrin et al. [15] conducted a randomized clinical trial (RCT) comparing free-handed flapped and fully-guided flapless implant placement surgery, replacing single missing teeth similar to the present study. The guided group results showed 2.53 ± 1.11 mm for the mean apical linear deviation (ALD) and 2.2 ± 1.1° for the mean angular deviation (AD). De Oliveira et al. [28] conducted the study with the largest sample size (115 implants) but on fully edentulous patients; by which mean ALD of 2.41 ± 0.74 mm and mean AD of 2.41 ± 0.15° for maxillary, and mean ALD of 2.18 ± 0.43 mm and mean AD of 2.50 ± 0.43° for mandibular implants were obtained. Valente et al. [21] also included a considerable sample size of 89 implants; subsequently, a mean ALD of 1.6 ± 1.2 mm and a mean AD of 7.9 ± 4.7° resulted. This study included partially and fully edentulous patients, meaning both tissue- and tooth-supported templates were used. Different data acquisition protocols, different analyzing methods, and inclusion of both tissue- and tooth-supported templated might be the reason between the current study and the mentioned studies’ results.

The introduction of intraoral and tabletop scanners has led to the next generation of virtual planning systems. These systems rely on the superimposition of CBCT and scan records, which consider soft tissue morphology in contrast with the conventional dual scan techniques. Moreover, these modern systems decrease clinical sessions and provide more flexibility as the procedure can be modified at any time to comply with different implant systems and designing software [26].

There are a few studies which calculated accuracy of implant placement using CBCT and intraoral scans [31–35]. The study with the largest sample size (145 fixtures) showed mean ALD of 1.06 ± 0.44 mm and mean AD of 2.72 ± 1.42°. Maximum numbers calculated for mean AD and mean ALD in the mentioned studies were 3.1 ± 2.3° [34] and 1.3 ± 0.6 mm [34], respectively. Minimum numbers were also 2.25 ± 1.41° [35] and 1.06 ± 0.44 mm [32]. Different surgical and prosthetic settings, analyzing methods, and case selection criteria (length of the edentulous space) might be responsible for the different results in the present study compared with the previous ones.

Little research has been conducted on immediate-loading protocols using prefabricated restorations in the context of fully-guided flapless implant surgery. Ko et al. [36] evaluated the success rate and the marginal bone loss of immediate and delayed loading protocols after a fully-guided flapless surgery. Even though the success rate was lower in the immediate loading group, marginal bone loss was acceptable in both groups. Oh et al. [19] have calculated the angular deviation of prefabricated screw-type provisional restorations after single-implant placement in an experimental study on typodonts, which lays grounds for further clinical studies. The present study showed that discrepancies between the virtually planned and delivered provisional restorations should be expected. Although, further studies are still needed to evaluate prefabricated restoration’s accuracy and success.

Regarding the current study’s limitations, it might be necessary to include a greater sample size in future studies to yield more solid results. Applying other surgical and prosthetic protocols, as well as using different intraoral scanners and computer designing programs, might help form a comprehensive opinion about the accuracy of digital implant placement. Finally, employing and comparing other calculation methods of the implant placement accuracy (using different software and protocols) in future studies might help understand each method’s errors and limitations.

## Conclusion

The implant placement accuracy using the DIONAVI protocol can be described by a mean angular deviation of 5.07° and a mean linear apical deviation of 1.74 mm, which might prove helpful to the clinician using it. Although these findings are in the higher range of the deviations calculated in the previous studies with different implant placement and calculation methods, further studies are required to reach a final verdict on the DIONAVI protocol’s accuracy. Regarding immediate-loading protocols and prefabricated provisional restorations, more research is vital to fully understand their pros and cons before arriving at a conclusion to apply them in routine care.

## Data Availability

The dataset used and/or analyzed during the current study are available from the corresponding author upon reasonable request.

## Abbreviations

AD: Angular deviation
ALD: Apical linear deviation
BOP: Bleeding on probing
CAD/CAM: Computer-aided design/computer-aided manufacturing
CBCT: Cone-beam computed tomography
DICOM: Digital Imaging and Communications in Medicine
RCT: Randomized clinical trial
STL: Standard Tessellation Language
